# Perseverance

**Published:** 1890-03-15

**Authors:** 


### Words of Consolation.

### PERSEVERANCE.

*For Reading to the Sick.*

Whether or not success will attend any undertaking depends entirely on how the details of the work are carried out. If we put our hearts into all we do, and are painstaking and industrious, we shall, notwithstanding checks and drawbacks, be triumphant in the end. But if on the contrary, we let our minds run on anything and everything but the matter in hand, we may safely say we are preparing for an utter failure. For instance, a child may determine to gain a prize at school; at the beginning of the term he works with all his might, but after a bit his energy slackens, his companions entice him to play, and soon the pleasure of the moment makes him forget the reward in the future, which he once so earnestly desired. Lessons are badly learned and said, he becomes careless and indifferent, and ends in disgrace at the bottom of his class. Again, many a man has begun life with every chance of doing well. With moderate care and attention he may get a good income, and if he work hard may become a partner in the business. This is the end he sets before him, but he must keep it constantly in view, or he will be slothful in his daily tasks, will put off to the morrow what ought to be done to-day, and take his pleasure and ease when he should be girding up his loins to bear the heat and burden of the day. Bad habits given way to always increase, and the end of his life finds him in no better position than that with which he started, and very probably a much worse one.

These instances are pictures of the way in which we are too apt to deal with our religious life, the life of the soul. We have been brought up carefully by a tender mother, and trained in the way in which we should go. When we begin to think for ourselves we form good resolutions to make the service of God our duty and pleasure, and to gain heaven is to be our aim and great reward. How long do these good resolutions last? Only while we pay strict attention to the little details of our conduct, while we are strictly honest and speak the truth in sincerity, and do to our neighbours as we would have them do to us. To most of the young these restrictions are tiresome. To them life seems endless; there is plenty of time to enjoy themselves, they say, and then we will begin to give our minds to another world, but they forget how the young as well as the aged and infirm are often taken away by death. We must work, therefore, and persevere in the work of our salvation while it is day, for the night cometh when none can work.

St. Paul, in writing to the Corinthians, likens the spiritual life to a race in which everyone strives to win an earthly crown, for which end they must be temperate in all things. We know, too, that in England those who desire to win in any sports have to train their bodies and keep them in subjection. In like manner the Apostle recommends us to be sober and watchful over our minds as well as our bodies, lest we should fail in that heavenly race set before us. And this keeping ourselves temperate requires constant perseverance.

